# Potential Effects of Phenolic Compounds That Can Be Found in Olive Oil on Wound Healing

**DOI:** 10.3390/foods10071642

**Published:** 2021-07-15

**Authors:** Lucia Melguizo-Rodríguez, Elvira de Luna-Bertos, Javier Ramos-Torrecillas, Rebeca Illescas-Montesa, Victor Javier Costela-Ruiz, Olga García-Martínez

**Affiliations:** 1 Biomedical Group (BIO277), Department of Nursing, Faculty of Health Sciences, University of Granada, Avda. Ilustración 60, 18016 Granada, Spain; luciamr@ugr.es (L.M.-R.); elviradlb@ugr.es (E.d.L.-B.); jrt@ugr.es (J.R.-T.); rebecaim@ugr.es (R.I.-M.); ogm@ugr.es (O.G.-M.); 2 Institute of Biosanitary Research, ibs.Granada, C/Doctor Azpitarte 4, 4^a^ planta, 18012 Granada, Spain

**Keywords:** extra virgin olive oil, phenolic compounds, wound healing, tissue regeneration

## Abstract

The treatment of tissue damage produced by physical, chemical, or mechanical agents involves considerable direct and indirect costs to health care systems. Wound healing involves a series of molecular and cellular events aimed at repairing the defect in tissue integrity. These events can be favored by various natural agents, including the polyphenols in extra virgin olive oil (EVOO). The objective of this study was to review data on the potential effects of different phenolic compounds that can also be found in EVOO on wound healing and closure. Results of in vitro and animal studies demonstrate that polyphenols from different plant species, also present in EVOO, participate in different aspects of wound healing, accelerating this process through their anti-inflammatory, antioxidant, and antimicrobial properties and their stimulation of angiogenic activities required for granulation tissue formation and wound re-epithelialization. These results indicate the potential usefulness of EVOO phenolic compounds for wound treatment, either alone or in combination with other therapies. Human studies are warranted to verify this proposition.

## 1. Introduction

The treatment of wounds represents a considerable economic burden for health care systems worldwide. In the USA, 8.2 million individuals covered by “Medicare” were treated for wounds in 2014, at an estimated cost ranging between $28.1 billion and $96.8 billion [1]. In the UK, it was estimated that the treatment of wounds and associated complications cost the National Health Service a total of £5.3 billion in 2012/13, with £18.6 million spent on practice nurse visits, £10.9 million on community nurse visits, £7.7 million on physician visits, and £3.4 million on hospital outpatient visits [2].

A wound is defined as a loss of skin integrity and function produced by physical, chemical, or mechanical agents [3]. Wounds are classified as acute, i.e., healing within 7–10 days, or chronic, i.e., failing to close within this time period after a torpid and disordered healing process. Wound chronicity has been associated with various factors, including underlying disease, infection, prolonged inflammation, consumption of medications, and aging-related oxidative stress [4,5,6].

Wound healing is characterized by a series of molecular and cellular events aimed at repairing the defect in tissue integrity. This process can give rise to regeneration, in which specific lost tissue is replaced with parenchymatous cells of the same type, and/or repair, in which the tissue is replaced by non-differentiated elements of connective and support tissue, resulting in a scar [7]. Cellular and biochemical events in wound healing can be divided into inflammatory, proliferative, and remodeling phases. The inflammatory phase involves intense vascular activity characterized by: exudate production; blood coagulation; hemostasis; the release by platelets of growth factors and proinflammatory cytokines that attract leukocytes to the lesioned area; and the activation of immune cells (mastocytes, gamma-delta T cells, and Langerhans cells) that release cytokines and chemotactic factors that attract different cell populations [8,9,10,11]. This is followed by the proliferative phase, aimed at wound angiogenesis, fibroplasia, and re-epithelialization [12]. It is characterized by the migration and proliferation of: endothelial cells, responsible for the formation of new vessels; fibroblasts, responsible for the production of collagen and other extracellular matrix components needed to form granulation tissue; and keratinocytes, responsible for the formation of epithelium over the wound surface [12,13,14]. In the final remodeling phase, scar maturation is promoted through the substitution of type III collagen, widely present in granulation tissue, by type I collagen (COL-I), characteristic of the human dermis [15]. 

The wound healing process can be favored by the antioxidant, antimicrobial, and/or biostimulatory properties of certain natural agents. Among these, the positive effect of polyphenols on tissue regeneration in some vegetable species is well documented. For instance, it has been found that resveratrol, widely present in grapes and red wine, can control oxidative stress, stimulating wound healing and diminishing the inflammatory response [16]. Proanthocyanidins in berries and curcumin in turmeric have also been found to shorten the inflammatory phase and favor wound healing by reducing the production of tumor necrosis factor-α (TNFα), interleukuin-1 (IL-1), and/or reactive oxygen species (ROS) [17,18]. Quercetin, a flavonoid present in peppers, tomatoes, apples, and oranges, also possesses anti-inflammatory, antioxidant, and antimicrobial properties and has the capacity to stimulate the production of collagen and the proliferation of fibroblasts and keratinocytes, among other cell populations [19]. Polyphenols in extra virgin olive oil (EVOO) have also demonstrated beneficial effects in wound healing. However, only limited evidence is available on the mechanisms underlying the effects of EVOO polyphenols on tissue regeneration [20,21,22,23]. The objective of this study was to provide an updated review of published data on the impact of different phenolic compounds that can also be found in EVOO on wound healing. 

## 2. Results

EVOO is the only olive oil obtained by pressing and one of the few that undergo no additional refining process. It comprises triacylglycerols (~98%), fatty acids, and mono- and diacylglycerols. More than 230 minor compounds, notably phenolic compounds, constitute around 2% of the total weight of EVOO. It contains five main classes of phenolic compounds: flavonoids, lignans, phenolic alcohols, and secoiridoids [24].

Flavonoids, secoiridoids, phenolic acids, and phenolic alcohols are the main polyphenols found in olives. Flavonoids, phenolic acids, and phenolic alcohols are present in numerous fruits and vegetables from various botanical families, whereas secoiridoids are only found in plants of the Olearacea family, including the olive tree (*Olea europaea* L.) [25]. The main findings of this review are summarized in Table 1.

### 2.1. Flavonoids

In general, flavonoids are released in EVOO at the end of the maturation phase due to hydrolytic processes [92].

#### 2.1.1. Rutin

The flavonoid with the highest concentration in EVOO is rutin [92,93], which has been attributed with anti-inflammatory, antioxidant, and neuro-, nephro-, and hepato-protective properties [94,95,96]. In an in vivo model of tissue regeneration, the administration of rutin-supplemented hydrogels reduced the wound area in rats, attributed to a decrease in oxidative stress markers (e.g., lipid peroxidation); the production of carbonyl protein through the oxidation of certain amino-acids, including lysine, proline, or arginine; and/or a treatment-induced increase in protein levels [26]. In another rat study, the intraperitoneal administration of rutin proved effective in the treatment of diabetic ulcers, reducing the wound area and increasing the percentage closure by stimulating the production of antioxidant enzymes and inhibiting the expression of metalloproteinases and vascular endothelial growth factor (VEGF), thereby lowering oxidative stress and inflammatory responses [27]. In an in vitro study, Pivec et al. [28] demonstrated that the water-based enzymatic polymerization of rutin enhances its antioxidant activity, promoting the viability of fibroblasts and HaCat cells (non-tumoral human keratinocytes), evidencing its potential usefulness in tissue regeneration and wound healing. The regenerative effect of rutin has also been related to epidermal growth factor-controlled cytoplasmic and nuclear pathways and closely associated with the activity of keratinocytes with an important role in the response of epidermal cells to wounds [29]. The authors reported a dose-dependent reduction in monocyte chemoattractant protein-1 and inducible protein-10 and increase in IL-8 after treating human keratinocytes with rutin.

#### 2.1.2. Luteolin 

Luteolin has anti-inflammatory, antioxidant, and cell-protective properties and is present in various vegetable species, including EVOO [97,98,99]. Luteolin is known to affect multiple cell functions and to raise levels of E-cadherin, an important molecule for binding certain cells responsible for the integrity of animal tissue [30,34]. In an in vitro study, the treatment of cultured human platelets with luteolin influenced their coagulation and fibrin polymer formation, inhibiting procoagulant biomolecules such as fibrin and factor X [31]. Luteolin also reduces the initial inflammatory phase of wound healing and may prevent prolonged inflammatory processes, avoiding new local damage and a delay in wound closure [100]. This flavonoid also reduces the migration of leukocytes, the loss of plasma and reduction of edema, which may be of benefit after the initial immune response to a wound [101]. Luteolin and its derivatives are known to increase the proliferation of fibroblasts and/or keratinocytes, thereby accelerating wound closure [32,33]. In later phases of healing, luteolin can inhibit the activity of collagenase and hyaluronidase, enhancing the stability of the extracellular matrix [35].

#### 2.1.3. Apigenin

Like the other polyphenols belonging to the flavonoid group, apigenin (Api) has been extensively studied for its antioxidant properties [102]. However, its benefits are not only limited to its ability to neutralise ROS, but it has also been shown to modulate wound healing and healing processes. In vivo assays by Süntar et al. showed that Api induced an improvement in wound healing, with faster re-epithelialisation and higher collagen concentration compared to the control [36]. It appears that this effect could be related to the anti-inflammatory activity of Api, which was determined by Whittle’s method. Furthermore, in vitro assays developed by the same authors showed that Api is able to inhibit collagenase and hyaluronidase enzymes at a dose of 100 µg/mL, thus corroborating its regenerative potential in wound healing. In other in vitro studies previously developed by our research group, it was observed that Api at a dose of 10^−6^ M i stimulates the growth and migration of human fibroblasts in culture, showing an antimicrobial effect by inhibiting the growth of microorganisms of diverse nature, such as *Staphylococcus aureus, Staphylococcus epidermidis, Escherichia coli, Proteus* spp., and *Candida albicans* [37]. These antibacterial effects have also been documented by Cheng et al. according to which Api is able to inhibit the haemolysis of rabbit erythrocytes caused by *S. aureus*. Similarly, the application of a gel of Api and lysin LysGH15 showed bactericidal capacity against *S. aureus* and methicillin-resistant Staphylococcus aureus. These authors observed not only an antimicrobial effect, but also that this gel reduced the levels of proinflammatory cytokines accelerating wound healing in a mouse model [38]. Other authors have also studied the effectiveness of using Api-based gels for wound healing. This is the case of Shukla et al., who tested the benefits of this compound by making and applying a chitosan-based gel enriched with Api in an in vivo diabetic wound model and in a dead space wound model. These authors showed that catalase and superoxide dismutase levels were elevated in the group treated with the enriched gel, in addition to a greater antioxidant effect and increased healing compared to the group that was only treated with the chitosan gel [39]. Other studies carried out on recreated wound models in mice have shown that the topical application of an Api-based gel accelerates re-epithelialisation, reduces inflammatory processes, and promotes neovascularisation of the injured tissue [40].

### 2.2. Secoiridoids

Secoiridoids are characterized by the presence of an elenolic acid derivative in their molecular structure. Along with lignans, they are the most abundant phenolic compounds in EVOO.

#### Oleuropein

Oleuropein (OLE), a phenolic compound found in olive leaves, olives, and EVOO, has important antioxidant and anti-inflammatory properties [103]. OLE intervenes in dermal regenerative bioprocesses during wound healing, playing an anti-inflammatory role by reducing oxidative stress and lipopolysaccharide (LPS)-induced cell death [41,104,105,106] 

In vitro analysis of the biological effect of a phenolic compound concentrate from Olive Mill Wastewater, rich in OLE, showed an increase of more than 70% in the viability of HaCaT cells at 24 and 48 h post-treatment. Scratch assay results evidenced a significant increase in HaCaTcell migration versus controls, obtaining an 80% reduction in wound area at 18 h and complete closure at 24 h [42]. In addition, OLE treatment of human umbilical vein endothelial cell cultures (HUVEC-C) increased cell viability in comparison to non-treated controls [41]. In in vivo mouse studies of wound healing, intradermal OLE injections favored re-epithelialization by increasing the number of collagen fibers and upregulating VEGF protein expression [43,44].

Spanish researchers have patented various pharmaceutical formulae (gel, cream [o/w emulsion], or aqueous solution) that contain OLE as the sole active ingredient. Their application on diabetic foot, vascular, and pressure ulcers (PU) was found to be more effective than conventional treatments at all tested concentrations, with no significant differences as a function of treatment dose or pharmaceutical formulation. The best outcomes were obtained at the highest concentration used (10^−2^ M) [45].

### 2.3. Phenolic Acids

Phenolic acids are secondary metabolites of aromatic plants. They are present in small amounts in EVOO and are associated with its sensory and organoleptic qualities [107].

#### 2.3.1. Gallic Acid

Gallic acid (GA) can be obtained by the hydrolytic decomposition of tannic acid using a glycoprotein esterase [24,108]. It has been found to have antioxidant, antimicrobial, anti-inflammatory, and anticarcinogenic capacities as well as protective gastric, cardiac, neurological, and dermal effects [46,109].

With respect to the role of GA in wound regeneration, in vitro studies of HaCaT keratinocytes, MEF mouse embryonic fibroblasts, and HF21 human fibroblast cells reported that GA directly regulates the expression of antioxidant genes and accelerates the migration of the studied cell populations under both physiologic and hyperglycemic conditions. GA also activates factors that play a relevant role in wound healing, such as focal adhesion kinases, c-Jun N-terminal kinases, and kinases regulated by extracellular signals [47]. GA has been found to inhibit the proliferation, migration, invasion, and cell cycle progression of fibroblasts isolated from keloids, promoting their apoptosis, and these effects are likely mediated by inhibition of the AKT/ERK signaling pathway [48]. Differences between the aforementioned studies in the functions attributed to GA may be explained by the distinct origins and characteristics of the cell lines under study. 

With regard to its antimicrobial activity, GA has proven capable of inhibiting the motility, adhesion, and biofilm formation of multiple microorganisms involved in wound contamination, including *Pseudomona aeruginosa* and *Staphylococcus aureus* [110]. GA treatment has also been found to alter the cell membrane integrity of Gram-positive and Gram-negative bacteria, compromising the permeability of the bacterial membrane and increasing the accumulation of antibiotics within the microorganism [46,49,111].

Prompted by the above findings, researchers have recently focused on GA formulations based on chitosan to enhance its properties and bioavailability. In this way, Kaparekar et al. [50] developed a collagen-fibrin scaffold with GA-loaded chitosan nanoparticles (GA-CSNPs) to favor the sustained release of GA in the wound bed and expand its in vitro and in vivo bioavailability. Results in cell lines and experimental rats showed that the GA-CSNP scaffold increases the cell migration rate, accelerating wound contraction and shortening the epithelialization phase. Sun et al. [51] obtained encouraging wound healing outcomes in rats by applying a biocompatible antibacterial dressing based on chitosan-copper-GA.

#### 2.3.2. Vanillic Acid

Vanillic acid (VA) possesses antioxidant, antimicrobial, and antibacterial properties [52,112,113,114,115,116] and is widely used in the food industry as an aromatic, additive, and preservative [117,118,119,120,121]. With respect to its potential for wound healing, observations of the effect of VA (among other phenolic compounds) on human dermal fibroblast and epidermal keratinocyte cell cultures indicated that it can improve the healing of chronic wounds (e.g., venous ulcers or burns), exerting a protective effect against the free radicals derived from this type of lesion [53].

#### 2.3.3. Caffeic Acid

Caffeic acid (CA), which has antimicrobial, anti-inflammatory, antioxidant, anxiolytic, and antitumor properties, is present in fruit, vegetables, tea, and red wine as well as EVOO [105,122].

In 2008, Song et al. [54] observed that the treatment of skin incisions with CA progressively increased the levels of collagen-like polymers. In their in vitro study of NIH 3T3 fibroblasts, CA was found to exert antioxidant and anti-inflammatory effects by inhibiting ROS generation and releasing arachidonic acid and prostaglandin 2 (PGE-2). These results were verified by Romana-Souza et al. [57] in an in vivo pressure ulcer model.

Fibroblast proliferation is a key event in wound healing. It was significantly increased by treatment with 10-6M CA for 24 h, which upregulated the expression of COL-I, VEGF, platelet-derived growth factor (PDGF), fibroblast growth factor (FGF), and transforming growth factor-β (TGFβ), stimulating the migration of fibroblasts at 24 h [37]. In another study, treatment with 0.5 mg/mL CA for 1 min accelerated the fibroblast proliferative response in 3T3-L1 mice at 24 h, although this response was inhibited when the mice were treated for 32 min, likely due to a cytotoxic effect [123].

CA derivatives such as phenyl ester have also been reported to favor the healing of skin wounds, acute lesions, and severe burns in animal studies, reducing the anti-inflammatory response and oxidative damage and stimulating wound contraction and re-epithelialization [58,124]. Similar results have been obtained in in vitro models [55,56,58].

#### 2.3.4. Ferulic Acid

Ferulic acid (FA), which has demonstrated elevated antioxidant and anti-inflammatory capacities and antidiabetic, antitumor, and neuro- and cardio-protective properties [125,126], is found in vegetables and cereals in free or conjugated form [24,127,128,129].

FA has been proposed to have therapeutic potential in different phases of wound healing [130]. In vitro studies have shown that FA increases angiogenesis and neovascularization by stimulating VEGF- and PDGF-mediated signaling pathways, increasing endothelial cell and fibroblast proliferation and migration and potentiating the repair response [37,59,60,61]. In other studies, the synergic action of FA associated to other molecules or essential oils with biostimulatory activity increased the viability, migration, and proliferation of fibroblasts and keratinocytes, among other cell types [62,131,132]. 

In vivo studies in diabetic rats confirmed the promotion of wound healing by FA treatment, which reduced the time to closure [62,63,64]. The combined treatment of incisions in rats using FA extract and other phenolic compounds from the flowering plant Boerhavia diffusa for 14 days reduced the wound area by 14% [133]. In a mouse study, the application of ointments containing FA and other plant extracts on induced burns modulated the inflammatory response and enhanced wound regeneration [134]. FA-supplemented hydrogels were recently developed to stimulate wound healing and reduce closure time, obtaining favorable outcomes in animal studies [135,136].

#### 2.3.5. P-Coumaric Acid 

p-coumaric acid (pCA), also known as 4-hydroxycinnamic acid, has been reported to have antioxidant, anti-inflammatory, antitumor, and antimicrobial properties and to exert preventive effects against neurologic, nephrological, and hepatic toxicity and UVB radiation [137,138]. pCA is a phenolic acid from the family of hydroxycinnamic acids and is found in broccoli, tomatoes, carrots, spinach, beans, and olives, among others [24,127,129,139,140].

Various in vitro studies have demonstrated the therapeutic potential of pCA for skin wound healing. Melguizo-Rodríguez et al. [37] found that treatment with 50 µM pCA had regenerative effects, stimulating the growth, differentiation, and migration of cultured human fibroblasts. Likewise, treatment of the murine fibroblast line 3T3 with 3 or 30 µM pCA enhanced wound closure in vitro and had no cytotoxic effects [65]. pCA, among other phenolic acids from plants, has also shown antioxidant activity on fibroblasts and keratinocytes, enhancing its wound healing capacity [53,140,141]. However, the benefits of pCA for wound healing have not yet been investigated in vivo.

### 2.4. Phenolic Alcohols

Hydroxytyrosol (HTyr) and tyrosol (Tyr) are the main phenolic alcohols in EVOO [142,143]. Their concentrations are generally low in fresh oil but rise during its storage [144].

#### 2.4.1. Hydroxytyrosol

HTyr has been reported to have antiatherogenic, cardioprotective [145,146], anticancer [147,148], neuroprotective [149,150], antimicrobial [151,152], anti-inflammatory, and antiplatelet properties [66,153]. It is released by OLE hydrolysis, which also gives rise to OLE aglycone and elenolic acid [154].

HTyr can modulate cell signaling and therefore play an important role in the healing process [155]. In in vitro studies, HTyr was found to inhibit the production of nitric oxide and PGE-2 and the expression of cyclooxygenase-2 (COX-2) and metalloproteinase-9 and to increase the production of TNF-α in monocytes [66]. HTyr treatment has promoted the proliferation and migration of human keratinocytes, porcine vascular endothelial cells, and HUVEC-C cells, thereby favoring angiogenesis and the re-epithelialization of skin wounds [67,68,69]. The antioxidant activity of HTyr was demonstrated in human keratinocytes subjected to UVB rays, observing that it protects against DNA damage and reduces intracellular ROS [70]. HTyr treatment was also found to reduce β-galactosidase, responsible for senescence, in dermal fibroblasts subjected to UVA light-induced photoaging, suggesting a possible anti-aging effect on skin [84].

Healing can be delayed by the biofilm present in wounds, and HTyr has demonstrated antimicrobial properties against different bacterial strains as well as antiviral activity. Therefore, treatment with HTyr could contribute to wound healing by diminishing the microbial load in the wound bed [71,72,156].

#### 2.4.2. Tyrosol

Tyr occurs as such or in the form of elenolic acid esters [157] and is distinguished by its ability to maintain its antioxidant capacity even under critical conditions [73]. Besides EVOO, it can be found in numerous foods (meats, nuts, vegetables, and fruit, etc.). 

Various in vitro studies have demonstrated the antioxidant and anti-inflammatory capacities of Tyr in mononuclear cells of human peripheral blood (lymphocytes, monocytes, and macrophages). Culture of these cell populations in an inflammatory environment and their subsequent treatment with Tyr significantly reduced the secretion of proinflammatory cytokines (IL-1β and macrophage migration inhibitory factor) and inhibited ROS production and the phosphorylation of mitogen-activated protein kinase, which may regulate inflammatory processes in chronic wounds [74]. The intravenous injection of Tyr in rats previously inoculated with LPS reduced their TNF-α, PGE-2, and nitrous oxide levels, inhibited their expression of COX-2, and produced a dose-dependent translocation of nuclear factor kappa-light-chain-enhancer of activated B cells [76].

With respect to vascularization of the wound bed, treatment of rats with Tyr for six weeks improved oxygen transport, reduced plasmatic viscosity, and favored the brain capillary network. The resulting improvements in blood pressure and circulation would promote the formation of granulation tissue in wounds [77].

Tyr, like HTyr, exerts antimicrobial activity. In an in vitro study, the growth of different *Escherichia coli* strains was inhibited by treatment with Tyr alone or combined with HTyr and OLE through the inhibition of ATP synthase. It can therefore be useful against local wound infections or the biofilm itself [75].

### 2.5. Lignans

Lignans are phenolic polymers which, together with tannins, contribute to the flavour, aroma and colour of the oil. The amount of lignans present in virgin olive oil can be up to 100 mg/kg, but there are considerable variations between different varieties of oil. These include pinoresinol and its derivatives [158].

#### Pinoresinol

With regard to pinoresinol, the literature on its effects on wound healing is still very limited. A recent study showed that pinoresinol is able to stimulate the proliferation and migration of keratinocytes in culture, enhancing the re-epithelialisation of cell-free zones in a dose-dependent manner with better results at 10 uM concentration [78]. These results have also been observed in other cell lines, such as fibroblasts. Thus, Do et al. demonstrated that pinoresinol treatment significantly stimulates the migration of mouse embryo fibroblasts, apparently due to the activation of Gi-coupled receptors, which are closely related to the migratory processes of the aforementioned cells [79]. In addition, the antimicrobial effect of this compound has also been documented, which would favour healing processes and complete wound closure [80,81].

### 2.6. Other Compounds

#### Vanillin

Vanillin is an aromatic aldehyde with antioxidant, anti-inflammatory, and antiapoptotic properties [84,85,86,87,88,159,160,161]. It is widely used in food, cosmetic, and pharmaceutical industries and is also present in EVOO [162,163,164].

With regard to its role in tissue regeneration and wound healing, a vanillin-enriched hydrogel was used to treat mesenchymal stem cells derived from the adipose tissue of diabetic rats and upregulated the expression of markers of vascular regeneration, collagen deposition, and hair follicle reconstruction [83]. In another study, the application of vanillin-supplemented chitosan membranes on skin incisions in diabetic rats reduced the wound size and TNF-α and IL-1β levels, increasing re-epithelialization, angiogenic stimulus, and collagen deposition [89]. Different formulations of vanillin have also shown bactericidal and/or bacteriostatic activity against Gram-positive and Gram-negative bacteria [82,90]. All of the aforementioned effects of vanillin have also been documented in burns [91].

## 3. Conclusions

The results of in vitro and in vivo studies support the favorable effect of EVOO polyphenols on the healing of skin lesions, attributable to their anti-inflammatory, antioxidant, antimicrobial, and angiogenic properties. These compounds represent an interesting therapeutic option for wound healing when applied alone, in combination with other treatments, or in phenolic extracts, which are rich in multiple polyphenols that may exert synergic effects. However, clinical trials are required to verify these findings in humans and further elucidate the mechanisms underlying the action of these molecules.

## Figures and Tables

**Table 1:** Summary of the role of phenolic compounds in wound healing.

EVOO Polyphenol Classification	Phenolic Compound	Clinical Relevance	Concentration Range	Reference
**FLAVONOIDS**	*Rutin*	In vitro Antioxidant and anti-inflammatory activity. Stimulation of cell viability of fibroblasts, HaCat cells and keratinocytes.	0.025 g (*w*/*w*)	[26]
			100 mg/kg body weight	[27]
		In vivo Reduction of wound area and increase in the rate of lesion closure through increased production of antioxidant enzymes lipid peroxidation or protein carbonyl peroxidation and decreased expression of oxidative stress markers and inflammatory processes.	0.5 g of rutin hydrate	[28]
			50 μM	[29]
	*Luteolin*	In vitro Elevates the expression of E-Cadherin which improve the generation of induced pluripotent stem cells. Inhibits procoagulant biomolecules Anti-oxidative and anti-inflammatory activities on keratinocytes and fibroblasts and immune cells Promotes fibroblast cells proliferation and migration.	7.5 μM	[30]
			25 μM	[31]
			Not specified	[32]
			1 μM	[33]
		In vivo Induces the expression of E-Cadherin and inhibits cancer metastases Faster wound healing.Reduction of edema and leukocyte migration in rats’ wounds. Inhibits hyaluronidase and collagenase activity in an in vivo wound healing assay.	20 μmol/L	[34]
			200 mg/kg	[35]
	*Apigenin*	In vitro Inhibition of collagenase and hyaluronidaseStimulates proliferation and migration of fibroblasts Antimicrobial properties	50 μg/mL	[36]
			10^−6^ M	[37]
			500 μg per 0.1 g ointment	[38]
		In vivo *Faster re-epithelialisation* *Increases levels of catalase and hyaluronidase* *Induces neovascularisation*	200 mg/kg	[36]
			500 μg per 0.1 g ointment	[38]
			30 mg	[39]
			0.2 g	[40]
**SECOIRIDOIDS**	*Oleuropein*	In vitro Increase of cell viability of HUVEC-C and HaCat cells and stimulation of migration of HaCat cells	50 μM	[41]
			1 and 0.1 mg/mL	[42]
		In vivo Anti-inflammatory effect. Promotes re-epithelialization by increasing collagen fibers and VEGF expression.	50 mg/kg	[43]
			50 mg/kg	[44]
		*Humans* More effective than conventional treatments in the management of diabetic foot, vascular and pressure ulcers	10^−1^ M–10^−11^ M	[45]
**PHENOLIC ACIDS**	*Gallic acid*	In vitro Antioxidant, anti-inflammatory and antimicrobial activity. Stimulation of cell proliferation and migration of fibroblasts (MET and HF21) and HaCat keratinocytes.	-	[46]
			10–200 μM	[47]
			1000 μM	[48]
			-	[49]
		In vivo Accelerates wound contraction and reduces epithelialization period combined with chitosan as a scaffold.	0.1–0.5 mg/mL	[50]
			40 mmol.L^−1^	[51]
	*Vanillic acid*	In vitro Antimicrobial effects Protection of cultured human skin cells against oxidative damage.	23–33 mM	[52]
			50, 100, 200, 500 μg/mL	[53]
	*Caffeic acid*	In vitro Antioxidant, anti-inflammatory and antimicrobial activity. Stimulation of cell proliferation, COL-1, VEGF, FGF, TGFβ and PDGF expression and migration of fibroblasts	10 mg/kg	[54]
			10^−6^ M	[37]
			10, 20, 30 µM	[55]
			10 μM	[56]
		In vivo Antioxidant, anti-inflammatory and antimicrobial activity. stimulation of wound contraction and re-epithelialization processes	10 mg/kg	[54]
			5 × 10^−6^ M	[57]
			10 μmol/kg	[58]
	*Ferulic acid*	In vitro Promotes the secretion of VEGF and PDGF and increases fibroblast proliferation and migration.	10^−6^ M	[37]
			10^−4^–10^−5^–10^−6^ M	[59]
			0.1–1–10 μg/mL	[60]
			10^−3^–10^−4^–10^−5^ M	[61]
		In vivo Reduces the healing time in diabetic rat wounds	1, 2, 5, 10 mg/mL	[62]
			10 mg/mL	[63]
			10 and 20 mg/kg	[64]
	*P-coumaric acid*	In vitro Stimulates growth, differentiation and migration of dermal fibroblasts and promotes wound closure of murine fibroblasts.	10^−6^ M	[37]
			30, 30 and 300 µM	[65]
**PHENOLIC** **ALCOHOLS**	*Hydroxytyrosol*	In vitro Antioxidant and anti-inflammatory activity. Promotes the proliferation of keratinocytes and endothelial cells, favoring the processes of angiogenesis and re-epithelialization. Antibacterial and antiviral activity	1–10 μmol/L	[66]
			0–100 µM	[67]
			1–5 µM	[68]
			10–100 µM	[69]
			25–100 µM	[70]
			1–10,000 µmol/L	[71]
			10–40 mg/Kg	[72]
	*Tyrosol*	In vitro Antioxidant and anti-inflammatory activity. Antibacterial activity	-	[73]
			0.5–1 µM	[74]
			0–35 mM	[75]
		In vivo Antioxidant and anti-inflammatory activity. Improves vascularization and circulation in rats	10–100 µM	[76]
			50 mg/kg daily i.g. for 6 weeks	[77]
**LIGNANS**	*Pinoresinol*	In vitro Stimulates proliferation and migration of keratinocytes and mouse embryo fibroblasts	10 µM	[78]
			10 µM	[79]
			15.2 and 10.9 µM	[80]
			100 µg and 2 mg/sensidisk	[81]
**OTHER** **COMPOUNDS**	*Vanillin*	In vitro Antimicrobial properties In vitro/in vivo Modulation of stem cells plasticity and therapeutic action in diabetic wound healing. Stimulates vascular regeneration, collagen deposition.	0.05 mol, 5.00 g	[82]
			25, 50, 70 µM	[83]
		In vivo Antimicrobial properties Protective effects against chronic mild stress induced in rats. Effective against bacteria in skin burns and promotion of epidermis regeneration and vascularization. Antimutagenic, antioxidant and anti-inflammatory properties. Antinociceptive properties in a visceral inflammatory model in mice. Reduce wound healing size, increase reepithelization, angiogenic stimulus, collagen deposition, reduction of levels of TNF-α, IL-1β, and increase of levels of IL-10, TNF-β and VEGF in diabetic rats.	40 mg/Kg	[84]
			10 and 40 mg/kg	[85]
			3.1–6.3–12.5–25 µM	[86]
			1–10 mg/Kg	[87]
			150 mg/kg	[88]
			192.2 mg, 126 mmol	[89]
			2.06 g, 10 mmol	[90]
			-	[91]

## Data Availability

Not applicable.

## Abbreviations

Tumor necrosis factor α: TNFα, interleukin 1: IL-1, Reactive oxygen species: ROS, extra-virgin olive oil: EVOO, Vascular Endothelial Growth Factor: VEGF, Oleuropein: OLE, Lipopolysaccharides: LPS, Human Umbilical Vein Endothelial Cells: HUVEC-C, pressure ulcers: PU, gallic acid: GA, vanillic acid: VA, caffeic acid: CA, Prostaglandin E2: PGE-2, collagen type I: COL-I, platelet derived growth factor: PDGF, Fibroblast growth factor: FGF, Transforming growth factor β: TGFβ, Ferulic acid: FA, Apigenin: Api, p-coumaric: pCA, hydroxytyrosol: HTyr, tyrosol: Tyr, ciclooxigenasa-2: COX-2.

## References

[1] Sen C.K. (2019). Human Wounds and Its Burden: An Updated Compendium of Estimates. Adv. Wound Care.

[2] Guest J.F., Ayoub N., McIlwraith T., Uchegbu I., Gerrish A., Weidlich D., Vowden K., Vowden P. (2015). Health economic burden that wounds impose on the National Health Service in the UK. BMJ Open.

[3] Imran H., Ahmad M., Rahman A., Yaqeen Z., Sohail T., Fatima N., Iqbal W., Yaqeen S.S. (2015). Evaluation of wound healing effects between Salvadora persica ointment and Solcoseryl jelly in animal model. Pak. J. Pharm. Sci..

[4] Baranoski S., Ayello E.A. (2008). Wound Care Essentials: Practice Principles.

[5] Farrar D. (2011). Advanced Wound Repair Therapies.

[6] Ibrahim N.I., Wong S.K., Mohamed I.N., Mohamed N., Chin K.Y., Ima-Nirwana S., Shuid A.N. (2018). Wound Healing Properties of Selected Natural Products. Int. J. Environ. Res. Public. Health.

[7] de Gonzalez A.C.O., Costa T.F., de Andrade Z.A., Medrado A.R.A.P. (2016). Wound healing—A literature review. An. Bras. Dermatol..

[8] Eming S.A., Krieg T., Davidson J.M. (2007). Inflammation in wound repair: Molecular and cellular mechanisms. J. Invest. Dermatol..

[9] Cumberbatch M., Dearman R.J., Griffiths C.E., Kimber I. (2000). Langerhans cell migration. Clin. Exp. Dermatol..

[10] Jameson J.M., Sharp L.L., Witherden D.A., Havran W.L. (2004). Regulation of skin cell homeostasis by gamma delta T cells. Front. Biosci. J. Virtual Libr..

[11] Noli C., Miolo A. (2001). The mast cell in wound healing. Vet. Dermatol..

[12] Li J., Chen J., Kirsner R. (2007). Pathophysiology of acute wound healing. Clin. Dermatol..

[13] Isaac C., de Ladeira P.R.S., do Rêgo F.M.P., Aldunate J.C.B., Ferreira M.C. (2010). Processo de cura das feridas: Cicatrização fisiológica. Rev. Med..

[14] Tonnesen M.G., Feng X., Clark R.A. (2000). Angiogenesis in wound healing. J. Investig. Dermatol. Symp. Proc..

[15] Shaw T.J., Martin P. (2009). Wound repair at a glance. J. Cell Sci..

[16] Zhou X., Ruan Q., Ye Z., Chu Z., Xi M., Li M., Hu W., Guo X., Yao P., Xie W. (2021). Resveratrol accelerates wound healing by attenuating oxidative stress-induced impairment of cell proliferation and migration. Burns J. Int. Soc. Burn Inj..

[17] Barchitta M., Maugeri A., Favara G., Magnano San Lio R., Evola G., Agodi A., Basile G. (2019). Nutrition and Wound Healing: An Overview Focusing on the Beneficial Effects of Curcumin. Int. J. Mol. Sci..

[18] Van de Velde F., Esposito D., Grace M.H., Pirovani M.E., Lila M.A. (2019). Anti-inflammatory and wound healing properties of polyphenolic extracts from strawberry and blackberry fruits. Food Res. Int. Ott. Ont.

[19] Polerà N., Badolato M., Perri F., Carullo G., Aiello F. (2019). Quercetin and its Natural Sources in Wound Healing Management. Curr. Med. Chem..

[20] Bayir Y., Un H., Ugan R.A., Akpinar E., Cadirci E., Calik I., Halici Z. (2019). The effects of Beeswax, Olive oil and Butter impregnated bandage on burn wound healing. Burns J. Int. Soc. Burn Inj..

[21] Donato-Trancoso A., Monte-Alto-Costa A., Romana-Souza B. (2016). Olive oil-induced reduction of oxidative damage and inflammation promotes wound healing of pressure ulcers in mice. J. Dermatol. Sci..

[22] Karimi Z., Behnammoghadam M., Rafiei H., Abdi N., Zoladl M., Talebianpoor M.S., Arya A., Khastavaneh M. (2019). Impact of olive oil and honey on healing of diabetic foot: A randomized controlled trial. Clin. Cosmet. Investig. Dermatol..

[23] Schanuel F.S., Saguie B.O., Monte-Alto-Costa A. (2019). Olive oil promotes wound healing of mice pressure injuries through NOS-2 and Nrf2. Appl. Physiol. Nutr. Metab. Physiol. Appl. Nutr. Metab..

[24] Servili M., Esposto S., Fabiani R., Urbani S., Taticchi A., Mariucci F., Selvaggini R., Montedoro G.F. (2009). Phenolic compounds in olive oil: Antioxidant, health and organoleptic activities according to their chemical structure. Inflammopharmacology.

[25] Han J., Talorete T.P.N., Yamada P., Isoda H. (2009). Anti-proliferative and apoptotic effects of oleuropein and hydroxytyrosol on human breast cancer MCF-7 cells. Cytotechnology.

[26] Almeida J.S., Benvegnú D.M., Boufleur N., Reckziegel P., Barcelos R.C.S., Coradini K., de Carvalho L.M., Bürger M.E., Beck R.C.R. (2012). Hydrogels containing rutin intended for cutaneous administration: Efficacy in wound healing in rats. Drug Dev. Ind. Pharm..

[27] Chen L.-Y., Huang C.-N., Liao C.-K., Chang H.-M., Kuan Y.-H., Tseng T.-J., Yen K.-J., Yang K.-L., Lin H.-C. (2020). Effects of Rutin on Wound Healing in Hyperglycemic Rats. Antioxidants.

[28] Pivec T., Kargl R., Maver U., Bračič M., Elschner T., Žagar E., Gradišnik L., Kleinschek K.S. (2019). Chemical Structure-Antioxidant Activity Relationship of Water-Based Enzymatic Polymerized Rutin and Its Wound Healing Potential. Polymers.

[29] Pastore S., Lulli D., Fidanza P., Potapovich A.I., Kostyuk V.A., De Luca C., Mikhal’Chik E., Korkina L.G. (2012). Plant Polyphenols Regulate Chemokine Expression and Tissue Repair in Human Keratinocytes Through Interaction with Cytoplasmic and Nuclear Components of Epidermal Growth Factor Receptor System. Antioxid. Redox Signal..

[30] Chen T., Yuan D., Wei B., Jiang J., Kang J., Ling K., Gu Y., Li J., Xiao L., Pei G. (2010). E-Cadherin-Mediated Cell–Cell Contact Is Critical for Induced Pluripotent Stem Cell Generation. Stem Cells.

[31] Guerrero J.A., Lozano M.L., Castillo J., Benavente-García O., Vicente V., Rivera J. (2005). Flavonoids inhibit platelet function through binding to the thromboxane A2 receptor. J. Thromb. Haemost. JTH.

[32] Bayrami Z., Khalighi-Sigaroodi F., Rahimi R., Farzaei M.H., Hodjat M., Baeeri M., Rahimifard M., Navaei-nigjeh M., Abdollahi M., Hajiaghaee R. (2017). In vitro wound healing activity of luteolin. Res. J. Pharmacogn..

[33] Wan D., Fu Y., Le Y., Zhang P., Ju J., Wang B., Zhang G., Wang Z., Su H., Wang L. (2019). Luteolin-7-glucoside Promotes Human Epidermal Stem Cell Proliferation by Upregulating β-Catenin, c-Myc, and Cyclin Expression. Stem Cells Int..

[34] Zhou Q., Yan B., Hu X., Li X.-B., Zhang J., Fang J. (2009). Luteolin inhibits invasion of prostate cancer PC3 cells through E-cadherin. Mol. Cancer Ther..

[35] Süntar I., Küpeli Akkol E., Keles H., Yesilada E., Sarker S.D., Arroo R., Baykal T. (2012). Efficacy of Daphne oleoides subsp. kurdica used for wound healing: Identification of active compounds through bioassay guided isolation technique. J. Ethnopharmacol..

[36] Süntar I., Küpeli Akkol E., Keles H., Yesilada E., Sarker S.D. (2013). Exploration of the wound healing potential of Helichrysum graveolens (Bieb.) Sweet: Isolation of apigenin as an active component. J. Ethnopharmacol..

[37] Melguizo-Rodríguez L., Illescas-Montes R., Costela-Ruiz V.J., Ramos-Torrecillas J., de Luna-Bertos E., García-Martínez O., Ruiz C. (2021). Antimicrobial properties of olive oil phenolic compounds and their regenerative capacity towards fibroblast cells. J. Tissue Viability.

[38] Cheng M., Zhang L., Zhang H., Li X., Wang Y., Xia F., Wang B., Cai R., Guo Z., Zhang Y. (2018). An Ointment Consisting of the Phage Lysin LysGH15 and Apigenin for Decolonization of Methicillin-Resistant Staphylococcus aureus from Skin Wounds. Viruses.

[39] Shukla R., Kashaw S.K., Jain A.P., Lodhi S. (2016). Fabrication of Apigenin loaded gellan gum–chitosan hydrogels (GGCH-HGs) for effective diabetic wound healing. Int. J. Biol. Macromol..

[40] Lopez-Jornet P., Camacho-Alonso F., Gómez-Garcia F., Molina Miñano F., Cañas X., Serafín A., Castillo J., Vicente-Ortega V. (2014). Effects of potassium apigenin and verbena extract on the wound healing process of SKH-1 mouse skin. Int. Wound J..

[41] Lo Vasco V.R., Leopizzi M., Di Maio V., Di Raimo T., Cesa S., Masci A., Rocca C.D. (2017). LPS, Oleuropein and Blueberry extracts affect the survival, morphology and Phosphoinositide signalling in stimulated human endothelial cells. J. Cell Commun. Signal..

[42] Alfano A., Corsuto L., Finamore R., Savarese M., Ferrara F., Falco S., Santabarbara G., De Rosa M., Schiraldi C. (2018). Valorization of Olive Mill Wastewater by Membrane Processes to Recover Natural Antioxidant Compounds for Cosmeceutical and Nutraceutical Applications or Functional Foods. Antioxidants.

[43] Mehraein F., Sarbishegi M., Aslani A. (2014). Evaluation of effect of oleuropein on skin wound healing in aged male BALB/c mice. Cell J..

[44] Mehraein F., Sarbishegi M., Aslani A. (2014). Therapeutic Effects of Oleuropein on Wounded Skin in Young Male Balb/c Mice. Wounds.

[45] Quesada-Gómez J.M.Q., Santiago-Mora R.M.S., Casado-Díaz A.C. (2017). Composiciones de Oleuropeína Para Cicatrización de Heridas y Úlceras en Ancianos y/o Diabéticos.

[46] Kahkeshani N., Farzaei F., Fotouhi M., Alavi S.S., Bahramsoltani R., Naseri R., Momtaz S., Abbasabadi Z., Rahimi R., Farzaei M.H. (2019). Pharmacological effects of gallic acid in health and diseases: A mechanistic review. Iran. J. Basic Med. Sci..

[47] Yang D.J., Moh S.H., Son D.H., You S., Kinyua A.W., Ko C.M., Song M., Yeo J., Choi Y.-H., Kim K.W. (2016). Gallic Acid Promotes Wound Healing in Normal and Hyperglucidic Conditions. Mol. Basel Switz..

[48] Wang X., Liu K., Ruan M., Yang J., Gao Z. (2018). Gallic acid inhibits fibroblast growth and migration in keloids through the AKT/ERK signaling pathway. Acta Biochim. Biophys. Sin..

[49] Teodoro G.R., Ellepola K., Seneviratne C.J., Koga-Ito C.Y. (2015). Potential Use of Phenolic Acids as Anti-Candida Agents: A Review. Front. Microbiol..

[50] Kaparekar P.S., Pathmanapan S., Anandasadagopan S.K. (2020). Polymeric scaffold of Gallic acid loaded chitosan nanoparticles infused with collagen-fibrin for wound dressing application. Int. J. Biol. Macromol..

[51] Sun X., Dong M., Guo Z., Zhang H., Wang J., Jia P., Bu T., Liu Y., Li L., Wang L. (2021). Multifunctional chitosan-copper-gallic acid based antibacterial nanocomposite wound dressing. Int. J. Biol. Macromol..

[52] Delaquis P., Stanich K., Toivonen P. (2005). Effect of pH on the inhibition of Listeria spp. by vanillin and vanillic acid. J. Food Prot..

[53] Phan T.T., Wang L., See P., Grayer R.J., Chan S.Y., Lee S.T. (2001). Phenolic compounds of Chromolaena odorata protect cultured skin cells from oxidative damage: Implication for cutaneous wound healing. Biol. Pharm. Bull..

[54] Song H.S., Park T.W., Sohn U.D., Shin Y.K., Choi B.C., Kim C.J., Sim S.S. (2008). The Effect of Caffeic Acid on Wound Healing in Skin-incised Mice. Korean J. Physiol. Pharmacol. Off. J. Korean Physiol. Soc. Korean Soc. Pharmacol..

[55] Li L., Sun W., Wu T., Lu R., Shi B. (2017). Caffeic acid phenethyl ester attenuates lipopolysaccharide-stimulated proinflammatory responses in human gingival fibroblasts via NF-κB and PI3K/Akt signaling pathway. Eur. J. Pharmacol..

[56] Lim K.-M., Bae S., Koo J.E., Kim E.-S., Bae O.-N., Lee J.Y. (2015). Suppression of skin inflammation in keratinocytes and acute/chronic disease models by caffeic acid phenethyl ester. Arch. Dermatol. Res..

[57] Romana-Souza B., Dos Santos J.S., Monte-Alto-Costa A. (2018). Caffeic acid phenethyl ester promotes wound healing of mice pressure ulcers affecting NF-κB, NOS2 and NRF2 expression. Life Sci..

[58] dos Santos J.S., Monte-Alto-Costa A. (2013). Caffeic acid phenethyl ester improves burn healing in rats through anti-inflammatory and antioxidant effects. J. Burn Care Res. Off. Publ. Am. Burn Assoc..

[59] Lin C.-M., Chiu J.-H., Wu I.-H., Wang B.-W., Pan C.-M., Chen Y.-H. (2010). Ferulic acid augments angiogenesis via VEGF, PDGF and HIF-1α. J. Nutr. Biochem..

[60] Wang J., Yuan Z., Zhao H., Ju D., Chen Y., Chen X., Zhang J. (2011). Ferulic acid promotes endothelial cells proliferation through up-regulating cyclin D1 and VEGF. J. Ethnopharmacol..

[61] San Miguel S.M., Opperman L.A., Allen E.P., Zielinski J., Svoboda K.K.H. (2011). Bioactive antioxidant mixtures promote proliferation and migration on human oral fibroblasts. Arch. Oral Biol..

[62] Poornima B., Korrapati P.S. (2017). Fabrication of chitosan-polycaprolactone composite nanofibrous scaffold for simultaneous delivery of ferulic acid and resveratrol. Carbohydr. Polym..

[63] Bairagi U., Mittal P., Singh J., Mishra B. (2018). Preparation, characterization, and in vivo evaluation of nano formulations of ferulic acid in diabetic wound healing. Drug Dev. Ind. Pharm..

[64] Ghaisas M.M., Kshirsagar S.B., Sahane R.S. (2014). Evaluation of wound healing activity of ferulic acid in diabetic rats. Int. Wound J..

[65] da Viana R.S., de Aquino F.L.T., Barreto E. (2020). Effect of trans-cinnamic acid and p-coumaric acid on fibroblast motility: A pilot comparative study of in silico lipophilicity measure. Nat. Prod. Res..

[66] Scoditti E., Nestola A., Massaro M., Calabriso N., Storelli C., De Caterina R., Carluccio M.A. (2014). Hydroxytyrosol suppresses MMP-9 and COX-2 activity and expression in activated human monocytes via PKCα and PKCβ1 inhibition. Atherosclerosis.

[67] Abate M., Citro M., Pisanti S., Caputo M., Martinelli R. (2021). Keratinocytes Migration Promotion, Proliferation Induction, and Free Radical Injury Prevention by 3-Hydroxytirosol. Int. J. Mol. Sci..

[68] Abate M., Pisanti S., Caputo M., Citro M., Vecchione C., Martinelli R. (2020). 3-Hydroxytyrosol Promotes Angiogenesis In Vitro by Stimulating Endothelial Cell Migration. Int. J. Mol. Sci..

[69] Zrelli H., Matsuoka M., Kitazaki S., Araki M., Kusunoki M., Zarrouk M., Miyazaki H. (2011). Hydroxytyrosol induces proliferation and cytoprotection against oxidative injury in vascular endothelial cells: Role of Nrf2 activation and HO-1 induction. J. Agric. Food Chem..

[70] Guo W., An Y., Jiang L., Geng C., Zhong L. (2010). The protective effects of hydroxytyrosol against UVB-induced DNA damage in HaCaT cells. Phytother. Res..

[71] Bedoya L.M., Beltrán M., Obregón-Calderón P., García-Pérez J., de la Torre H.E., González N., Pérez-Olmeda M., Auñón D., Capa L., Gómez-Acebo E. (2016). Hydroxytyrosol: A new class of microbicide displaying broad anti-HIV-1 activity. AIDS Lond. Engl..

[72] Wu H., Jiang K., Zhang T., Zhao G., Deng G. (2017). Hydroxytyrosol exerts an anti-inflammatory effect by suppressing Toll-like receptor 2 and TLR 2 downstream pathways in Staphylococcus aureus-induced mastitis in mice. J. Funct. Foods.

[73] Karković Marković A., Torić J., Barbarić M., Jakobušić Brala C. (2019). Hydroxytyrosol, Tyrosol and Derivatives and Their Potential Effects on Human Health. Molecules.

[74] Serra G., Deiana M., Spencer J.P.E., Corona G. (2017). Olive Oil Phenolics Prevent Oxysterol-Induced Proinflammatory Cytokine Secretion and Reactive Oxygen Species Production in Human Peripheral Blood Mononuclear Cells, Through Modulation of p38 and JNK Pathways. Mol. Nutr. Food Res..

[75] Amini A., Liu M., Ahmad Z. (2017). Understanding the link between antimicrobial properties of dietary olive phenolics and bacterial ATP synthase. Int. J. Biol. Macromol..

[76] Sato K., Mihara Y., Kanai K., Yamashita Y., Kimura Y., Itoh N. (2016). Tyrosol ameliorates lipopolysaccharide-induced ocular inflammation in rats via inhibition of nuclear factor (NF)-κB activation. J. Vet. Med. Sci..

[77] Plotnikov M.B., Aliev O.I., Sidekhmenova A.V., Shamanaev A.Y., Anishchenko A.M., Fomina T.I., Plotnikova T.M., Arkhipov A.M. (2018). Effect of p-tyrosol on hemorheological parameters and cerebral capillary network in young spontaneously hypertensive rats. Microvasc. Res..

[78] Goels T., Eichenauer E., Langeder J., Hoeller F., Sykora C., Tahir A., Urban E., Heiss E.H., Saukel J., Glasl S. (2020). Norway Spruce Balm: Phytochemical Composition and Ability to Enhance Re-epithelialization In Vitro. Planta Med..

[79] Do K.H., Choi Y.W., Kim E.K., Yun S.J., Kim M.S., Lee S.Y., Ha J.M., Kim J.H., Kim C.D., Son B.G. (2009). Pinoresinol-4,4’-di-O-beta-D-glucoside from Valeriana officinalis root stimulates calcium mobilization and chemotactic migration of mouse embryo fibroblasts. Phytomedicine Int. J. Phytother. Phytopharm..

[80] Ribeiro V.P., Arruda C., Mejia J.A.A., Bastos J., Tripathi S.K., Khan S.I., Khan I.A., Ali Z. (2021). Phytochemical, antiplasmodial, cytotoxic and antimicrobial evaluation of a Southeast Brazilian Brown Propolis produced by Apis mellifera bees. Chem. Biodivers..

[81] Céspedes C.L., Avila J.G., García A.M., Becerra J., Flores C., Aqueveque P., Bittner M., Hoeneisen M., Martinez M., Silva M. (2006). Antifungal and antibacterial activities of Araucaria araucana (Mol.) K. Koch heartwood lignans. Z. Naturforschung C J. Biosci..

[82] Yadav N., Monisha M., Niranjan R., Dubey A., Patil S., Priyadarshini R., Lochab B. (2021). Antibacterial performance of fully biobased chitosan-grafted-polybenzoxazine films: Elaboration and properties of released material. Carbohydr. Polym..

[83] Jin X., Shang Y., Zou Y., Xiao M., Huang H., Zhu S., Liu N., Li J., Wang W., Zhu P. (2020). Injectable Hypoxia-Induced Conductive Hydrogel to Promote Diabetic Wound Healing. ACS Appl. Mater. Interfaces.

[84] Lee J.-C., Kim I.H., Cho J.H., Lee T.-K., Park J.H., Ahn J.H., Shin B.N., Yan B.C., Kim J.-D., Jeon Y.H. (2018). Vanillin improves scopolamine-induced memory impairment through restoration of ID1 expression in the mouse hippocampus. Mol. Med. Rep..

[85] Abo-youssef A.M. (2016). Possible antidepressant effects of vanillin against experimentally induced chronic mild stress in rats. Beni-Suef Univ. J. Basic Appl. Sci..

[86] Tai A., Sawano T., Yazama F., Ito H. (2011). Evaluation of antioxidant activity of vanillin by using multiple antioxidant assays. Biochim. Biophys. Acta BBA Gen. Subj..

[87] Park S.-H., Sim Y.-B., Choi S.-M., Seo Y.-J., Kwon M.-S., Lee J.-K., Suh H.-W. (2009). Antinociceptive profiles and mechanisms of orally administered vanillin in the mice. Arch. Pharm. Res..

[88] Makni M., Chtourou Y., Fetoui H., Garoui E.M., Boudawara T., Zeghal N. (2011). Evaluation of the antioxidant, anti-inflammatory and hepatoprotective properties of vanillin in carbon tetrachloride-treated rats. Eur. J. Pharmacol..

[89] de Aragão Tavares E., de Medeiros W.M.T.Q., de Assis Pontes T.P., Barbosa M.M., de Araújo A.A., de Araújo R.F., Figueiredo J.G., Leitão R.C., da Silva Martins C., da Silva F.O.N. (2018). Chitosan Membrane Modified With a New Zinc(II)-Vanillin Complex Improves Skin Wound Healing in Diabetic Rats. Front. Pharmacol..

[90] Tian Y., Pang L., Zhang R., Xu T., Wang S., Yu B., Gao L., Cong H., Shen Y. (2020). Poly-tetrahydropyrimidine Antibacterial Hydrogel with Injectability and Self-Healing Ability for Curing the Purulent Subcutaneous Infection. ACS Appl. Mater. Interfaces.

[91] Zhou G., Ruhan A., Ge H., Wang L., Liu M., Wang B., Su H., Yan M., Xi Y., Fan Y. (2014). Research on a novel poly (vinyl alcohol)/lysine/vanillin wound dressing: Biocompatibility, bioactivity and antimicrobial activity. Burns J. Int. Soc. Burn Inj..

[92] Vinha A.F., Ferreres F., Silva B.M., Valentão P., Gonçalves A., Pereira J.A., Oliveira M.B., Seabra R.M., Andrade P.B. (2005). Phenolic profiles of Portuguese olive fruits (*Olea europaea* L.): Influences of cultivar and geographical origin. Food Chem..

[93] Gómez-Rico A., Fregapane G., Salvador M.D. (2008). Effect of cultivar and ripening on minor components in Spanish olive fruits and their corresponding virgin olive oils. Food Res. Int..

[94] Budzynska B., Faggio C., Kruk-Slomka M., Samec D., Nabavi S.F., Sureda A., Devi K.P., Nabavi S.M. (2019). Rutin as Neuroprotective Agent: From Bench to Bedside. Curr. Med. Chem..

[95] Ganeshpurkar A., Saluja A.K. (2017). The Pharmacological Potential of Rutin. Saudi Pharm. J. SPJ Off. Publ. Saudi Pharm. Soc..

[96] Song K., Na J.-Y., Kim S., Kwon J. (2015). Rutin upregulates neurotrophic factors resulting in attenuation of ethanol-induced oxidative stress in HT22 hippocampal neuronal cells. J. Sci. Food Agric..

[97] Yashin A., Yashin Y., Xia X., Nemzer B. (2017). Antioxidant Activity of Spices and Their Impact on Human Health: A Review. Antioxidants.

[98] Seelinger G., Merfort I., Schempp C.M. (2008). Anti-oxidant, anti-inflammatory and anti-allergic activities of luteolin. Planta Med..

[99] Neuhouser M.L. (2004). Dietary flavonoids and cancer risk: Evidence from human population studies. Nutr. Cancer.

[100] Guo Y.-F., Xu N.-N., Sun W., Zhao Y., Li C.-Y., Guo M.-Y. (2017). Luteolin reduces inflammation in Staphylococcus aureus-induced mastitis by inhibiting NF-kB activation and MMPs expression. Oncotarget.

[101] Fedel-Miyasato L.E.S., Kassuya C.A.L., Auharek S.A., Formagio A.S.N., Cardoso C.A.L., Mauro M.O., Cunha-Laura A.L., Monreal A.C.D., Vieira M.C., Oliveira R.J. (2014). Evaluation of anti-inflammatory, immunomodulatory, chemopreventive and wound healing potentials from Schinus terebinthifolius methanolic extract. Rev. Bras. Farmacogn..

[102] Mainka M., Czerwińska M.E., Osińska E., Ziaja M., Bazylko A. (2021). Screening of Antioxidative Properties and Inhibition of Inflammation-Linked Enzymes by Aqueous and Ethanolic Extracts of Plants Traditionally Used in Wound Healing in Poland. Antioxidants.

[103] Motawea M.H., Abd Elmaksoud H.A., Elharrif M.G., Desoky A.A.E., Ibrahimi A. (2020). Evaluation of Anti-inflammatory and Antioxidant Profile of Oleuropein in Experimentally Induced Ulcerative Colitis. Int. J. Mol. Cell. Med..

[104] Barbaro B., Toietta G., Maggio R., Arciello M., Tarocchi M., Galli A., Balsano C. (2014). Effects of the olive-derived polyphenol oleuropein on human health. Int. J. Mol. Sci..

[105] Gorzynik-Debicka M., Przychodzen P., Cappello F., Kuban-Jankowska A., Marino Gammazza A., Knap N., Wozniak M., Gorska-Ponikowska M. (2018). Potential Health Benefits of Olive Oil and Plant Polyphenols. Int. J. Mol. Sci..

[106] Hassen I., Casabianca H., Hosni K. (2014). Biological activities of the natural antioxidant oleuropein: Exceeding the expectation—A mini-review. J. Funct. Foods.

[107] Garrido Fernandez A., Adams M.R., Fernandez-Diez M.J. (1997). Table Olives—Production and Processing.

[108] Fernandes F.H.A., Salgado H.R.N. (2016). Gallic Acid: Review of the Methods of Determination and Quantification. Crit. Rev. Anal. Chem..

[109] Choubey S., Varughese L.R., Kumar V., Beniwal V. (2015). Medicinal importance of gallic acid and its ester derivatives: A patent review. Pharm. Pat. Anal..

[110] Shao D., Li J., Li J., Tang R., Liu L., Shi J., Huang Q., Yang H. (2015). Inhibition of Gallic Acid on the Growth and Biofilm Formation of Escherichia coli and Streptococcus mutans. J. Food Sci..

[111] Oh E., Jeon B. (2015). Synergistic anti-Campylobacter jejuni activity of fluoroquinolone and macrolide antibiotics with phenolic compounds. Front. Microbiol..

[112] Aziz N.H., Farag S.E., Mousa L.A., Abo-Zaid M.A. (1998). Comparative antibacterial and antifungal effects of some phenolic compounds. Microbios.

[113] Liu R.H. (2004). Potential synergy of phytochemicals in cancer prevention: Mechanism of action. J. Nutr..

[114] Ghanbari R., Anwar F., Alkharfy K.M., Gilani A.-H., Saari N. (2012). Valuable nutrients and functional bioactives in different parts of olive (*Olea europaea* L.)—A review. Int. J. Mol. Sci..

[115] Kakkar S., Bais S. (2014). A review on protocatechuic Acid and its pharmacological potential. ISRN Pharmacol..

[116] Juturu V., Watson R.R., Preedy V.R., Zibadi S. (2014). Chapter 82—Polyphenols and Cardiometabolic Syndrome. Polyphenols in Human Health and Disease.

[117] Zheng W., Wang S.Y. (2001). Antioxidant activity and phenolic compounds in selected herbs. J. Agric. Food Chem..

[118] Russell W.R., Scobbie L., Labat A., Duthie G.G. (2009). Selective bio-availability of phenolic acids from Scottish strawberries. Mol. Nutr. Food Res..

[119] Gitzinger M., Kemmer C., Fluri D.A., Daoud El-Baba M., Weber W., Fussenegger M. (2012). The food additive vanillic acid controls transgene expression in mammalian cells and mice. Nucleic Acids Res..

[120] Jun H.-I., Song G.-S., Yang E.-I., Youn Y., Kim Y.-S. (2012). Antioxidant activities and phenolic compounds of pigmented rice bran extracts. J. Food Sci..

[121] Palafox-Carlos H., Yahia E.M., González-Aguilar G.A. (2012). Identification and quantification of major phenolic compounds from mango (Mangifera indica, cv. Ataulfo) fruit by HPLC–DAD–MS/MS-ESI and their individual contribution to the antioxidant activity during ripening. Food Chem..

[122] Son S., Lewis B.A. (2002). Free radical scavenging and antioxidative activity of caffeic acid amide and ester analogues: Structure-activity relationship. J. Agric. Food Chem..

[123] Tsuruya M., Niwano Y., Nakamura K., Kanno T., Nakashima T., Egusa H., Sasaki K. (2014). Acceleration of Proliferative Response of Mouse Fibroblasts by Short-Time Pretreatment with Polyphenols. Appl. Biochem. Biotechnol..

[124] Serarslan G., Altuğ E., Kontas T., Atik E., Avci G. (2007). Caffeic acid phenethyl ester accelerates cutaneous wound healing in a rat model and decreases oxidative stress. Clin. Exp. Dermatol..

[125] Chaudhary A., Jaswal V.S., Choudhary S., Sharma A., Beniwal V., Tuli H.S., Sharma S. (2019). Ferulic Acid: A Promising Therapeutic Phytochemical and Recent Patents Advances. Recent Pat. Inflamm. Allergy Drug Discov..

[126] Budak N.H., Aykin E., Seydim A.C., Greene A.K., Guzel-Seydim Z.B. (2014). Functional properties of vinegar. J. Food Sci..

[127] Bayram B., Esatbeyoglu T., Schulze N., Ozcelik B., Frank J., Rimbach G. (2012). Comprehensive Analysis of Polyphenols in 55 Extra Virgin Olive Oils by HPLC-ECD and Their Correlation with Antioxidant Activities. Plant Foods Hum. Nutr..

[128] Manach C., Scalbert A., Morand C., Rémésy C., Jiménez L. (2004). Polyphenols: Food sources and bioavailability. Am. J. Clin. Nutr..

[129] Rashmi H.B., Negi P.S. (2020). Phenolic acids from vegetables: A review on processing stability and health benefits. Food Res. Int..

[130] Zduńska K., Dana A., Kolodziejczak A., Rotsztejn H. (2018). Antioxidant Properties of Ferulic Acid and Its Possible Application. Skin Pharmacol. Physiol..

[131] Carbone C., Caddeo C., Grimaudo M.A., Manno D.E., Serra A., Musumeci T. (2020). Ferulic Acid-NLC with Lavandula Essential Oil: A Possible Strategy for Wound-Healing?. Nanomaterials.

[132] Valacchi G., Grisci G., Sticozzi C., Lim Y., Paolino M., Giuliani G., Mendichi R., Belmonte G., Artusi R., Zanardi A. (2015). Wound healing properties of hyaluronan derivatives bearing ferulate residues. J. Mater. Chem. B.

[133] Juneja K., Mishra R., Chauhan S., Gupta S., Roy P., Sircar D. (2020). Metabolite profiling and wound-healing activity of Boerhavia diffusa leaf extracts using in vitro and in vivo models. J. Tradit. Complement. Med..

[134] Song Y., Zeng R., Hu L., Maffucci K.G., Ren X., Qu Y. (2017). In vivo wound healing and in vitro antioxidant activities of Bletilla striata phenolic extracts. Biomed. Pharmacother. Biomedecine Pharmacother..

[135] Tsai C.-Y., Woung L.-C., Yen J.-C., Tseng P.-C., Chiou S.-H., Sung Y.-J., Liu K.-T., Cheng Y.-H. (2016). Thermosensitive chitosan-based hydrogels for sustained release of ferulic acid on corneal wound healing. Carbohydr. Polym..

[136] Wei Q., Duan J., Ma G., Zhang W., Wang Q., Hu Z. (2019). Enzymatic crosslinking to fabricate antioxidant peptide-based supramolecular hydrogel for improving cutaneous wound healing. J. Mater. Chem. B.

[137] Ferreira P.S., Victorelli F.D., Fonseca-Santos B., Chorilli M. (2019). A Review of Analytical Methods for p-Coumaric Acid in Plant-Based Products, Beverages, and Biological Matrices. Crit. Rev. Anal. Chem..

[138] Seok J.K., Boo Y.C. (2015). p-Coumaric Acid Attenuates UVB-Induced Release of Stratifin from Keratinocytes and Indirectly Regulates Matrix Metalloproteinase 1 Release from Fibroblasts. Korean J. Physiol. Pharmacol. Off. J. Korean Physiol. Soc. Korean Soc. Pharmacol..

[139] El-Seedi H.R., El-Said A.M.A., Khalifa S.A.M., Göransson U., Bohlin L., Borg-Karlson A.-K., Verpoorte R. (2012). Biosynthesis, Natural Sources, Dietary Intake, Pharmacokinetic Properties, and Biological Activities of Hydroxycinnamic Acids. J. Agric. Food Chem..

[140] Sytar O., Hemmerich I., Zivcak M., Rauh C., Brestic M. (2018). Comparative analysis of bioactive phenolic compounds composition from 26 medicinal plants. Saudi J. Biol. Sci..

[141] Sowa I., Paduch R., Strzemski M., Zielińska S., Rydzik-Strzemska E., Sawicki J., Kocjan R., Polkowski J., Matkowski A., Latalski M. (2018). Proliferative and antioxidant activity of Symphytum officinale root extract. Nat. Prod. Res..

[142] Bonoli M., Montanucci M., Gallina Toschi T., Lercker G. (2003). Fast separation and determination of tyrosol, hydroxytyrosol and other phenolic compounds in extra-virgin olive oil by capillary zone electrophoresis with ultraviolet-diode array detection. J. Chromatogr. A.

[143] Bonoli M., Bendini A., Cerretani L., Lercker G., Toschi T.G. (2004). Qualitative and semiquantitative analysis of phenolic compounds in extra virgin olive oils as a function of the ripening degree of olive fruits by different analytical techniques. J. Agric. Food Chem..

[144] Montedoro G., Servili M., Baldioli M., Miniati E. (1992). Simple and hydrolyzable phenolic compounds in virgin olive oil. 1. Their extraction, separation, and quantitative and semiquantitative evaluation by HPLC. J. Agric. Food Chem..

[145] Catalán Ú., López de Las Hazas M.-C., Rubió L., Fernández-Castillejo S., Pedret A., de la Torre R., Motilva M.-J., Solà R. (2015). Protective effect of hydroxytyrosol and its predominant plasmatic human metabolites against endothelial dysfunction in human aortic endothelial cells. Mol. Nutr. Food Res..

[146] Perona J.S., Cabello-Moruno R., Ruiz-Gutierrez V. (2006). The role of virgin olive oil components in the modulation of endothelial function. J. Nutr. Biochem..

[147] Bernini R., Carastro I., Palmini G., Tanini A., Zonefrati R., Pinelli P., Brandi M.L., Romani A. (2017). Lipophilization of Hydroxytyrosol-Enriched Fractions from Olea europaea L. Byproducts and Evaluation of the in Vitro Effects on a Model of Colorectal Cancer Cells. J. Agric. Food Chem..

[148] Fabiani R., Sepporta M.V., Rosignoli P., De Bartolomeo A., Crescimanno M., Morozzi G. (2012). Anti-proliferative and pro-apoptotic activities of hydroxytyrosol on different tumour cells: The role of extracellular production of hydrogen peroxide. Eur. J. Nutr..

[149] Kamil K., Yazid M.D., Idrus R.B.H., Kumar J. (2020). Hydroxytyrosol Promotes Proliferation of Human Schwann Cells: An In Vitro Study. Int. J. Environ. Res. Public. Health.

[150] Rodríguez-Morató J., Xicota L., Fitó M., Farré M., Dierssen M., De la Torre R. (2015). Potential Role of Olive Oil Phenolic Compounds in the Prevention of Neurodegenerative Diseases. Molecules.

[151] Diallinas G., Rafailidou N., Kalpaktsi I., Komianou A.C., Tsouvali V., Zantza I., Mikros E., Skaltsounis A.L., Kostakis I.K. (2018). Hydroxytyrosol (HT) Analogs Act as Potent Antifungals by Direct Disruption of the Fungal Cell Membrane. Front. Microbiol..

[152] Yamada K., Ogawa H., Hara A., Yoshida Y., Yonezawa Y., Karibe K., Nghia V.B., Yoshimura H., Yamamoto Y., Yamada M. (2009). Mechanism of the antiviral effect of hydroxytyrosol on influenza virus appears to involve morphological change of the virus. Antivir. Res..

[153] Calabriso N., Gnoni A., Stanca E., Cavallo A., Damiano F., Siculella L., Carluccio M.A. (2018). Hydroxytyrosol Ameliorates Endothelial Function under Inflammatory Conditions by Preventing Mitochondrial Dysfunction. Oxid. Med. Cell. Longev..

[154] Granados-Principal S., Quiles J.L., Ramirez-Tortosa C.L., Sanchez-Rovira P., Ramirez-Tortosa M.C. (2010). Hydroxytyrosol: From laboratory investigations to future clinical trials. Nutr. Rev..

[155] Utami N.D., Nordin A., Katas H., Bt Hj Idrus R., Fauzi M.B. (2020). Molecular Action of Hydroxytyrosol in Wound Healing: An In Vitro Evidence-Based Review. Biomolecules.

[156] Bisignano G., Tomaino A., Lo Cascio R., Crisafi G., Uccella N., Saija A. (1999). On the in-vitro antimicrobial activity of oleuropein and hydroxytyrosol. J. Pharm. Pharmacol..

[157] Tuck K.L., Hayball P.J. (2002). Major phenolic compounds in olive oil: Metabolism and health effects. J. Nutr. Biochem..

[158] Brenes M., Hidalgo F.J., García A., Rios J.J., García P., Zamora R., Garrido A. (2000). Pinoresinol and 1-acetoxypinoresinol, two new phenolic compounds identified in olive oil. J. Am. Oil Chem. Soc..

[159] Kim Y., Florio S., Wang Q. (2017). Blast Analysis of Aging Transportation Structures with Little Stand-Off Distance. Congr. Tech. Adv..

[160] Shaughnessy D.T., Setzer R.W., DeMarini D.M. (2001). The antimutagenic effect of vanillin and cinnamaldehyde on spontaneous mutation in Salmonella TA104 is due to a reduction in mutations at GC but not AT sites. Mutat. Res. Mol. Mech. Mutagen..

[161] De Flora S., Bennicelli C., Rovida A., Scatolini L., Camoirano A. (1994). Inhibition of the ‘spontaneous’ mutagenicity in Salmonella typhimurium TA102 and TA104. Mutat. Res. Mol. Mech. Mutagen..

[162] Ho K., Yazan L.S., Ismail N., Ismail M. (2009). Apoptosis and cell cycle arrest of human colorectal cancer cell line HT-29 induced by vanillin. Cancer Epidemiol..

[163] Ho K., Yazan L.S., Ismail N., Ismail M. (2011). Toxicology study of vanillin on rats via oral and intra-peritoneal administration. Food Chem. Toxicol..

[164] Banerjee G., Chattopadhyay P. (2019). Vanillin biotechnology: The perspectives and future. J. Sci. Food Agric..

